# Velocities of Bone Mineral Accrual in Black and White American Children

**DOI:** 10.1002/jbmr.43

**Published:** 2010-01-29

**Authors:** Siu L Hui, Anthony J Perkins, Jaroslaw Harezlak, Munro Peacock, Cindy L McClintock, C Conrad Johnston

**Affiliations:** 1 Department of Medicine, Indiana University School of MedicineIndianapolis, IN, USA; 2 Regenstrief Institute, Inc., and Indiana University Center on Aging ResearchIndianapolis, IN, USA

**Keywords:** racial, bone density, growth

## Abstract

Black adults have higher bone mass than whites in the United States, but it is not clear when black children gain bone mineral faster than white children. We performed a cohort study to compare the growth velocity of total-body bone mineral content (TBMC) between black and white children of the same sex at different ages and stages of sexual maturity. TBMC and total-body area were measured in a cohort of 188 black and white boys and girls aged 5 to 15 years annually for up to 4 years. Rates of change in TBMC and area were found to vary with age and with Tanner stage. For both TBMC and area, growth velocities between black and white children differed significantly across Tanner stages. Age-specific velocities were higher in black children during prepuberty and initial entry into puberty but reversed in subsequent Tanner stages. Despite earlier entry into each Tanner stage, black children spent only an average of only 0.2 year longer in Tanner stages II through IV, and total gain in TBMC from age 5 to 15 was not higher in whites. In conclusion, the higher bone mass in black adults compared with whites cannot be attributed to faster accrual during puberty. It is due to black children's higher rate of bone mineral accrual in prepuberty and plausibly in postpuberty. Most of the racial difference in TBMC velocity can be explained by growth in size. © 2010 American Society for Bone and Mineral Research.

## Introduction

African Americans have higher bone mass and lower fracture rates than European Americans of the same sex and age.(1,2) The higher bone mass in older black adults is a result of their higher peak bone mass in early adulthood(3–5) and lower rates of loss with aging.(6,7) The racial difference in bone mineral content (BMC) and areal bone mineral density (aBMD = BMC/area), though not apparent in infancy,(8) emerges by early childhood and persists through adolescence and young adulthood.(3,8–14) It is unclear, however, when the greatest difference in the rate of skeletal mineral accrual occurs during the course of acquisition from less than 200 g of BMC in infancy(8) to more than 2000 g in adulthood.(10) Because a large proportion of the cumulative mineral acquisition occurs during the peripubertal years,(15) we hypothesized that much of the higher BMC in black adults is gained through higher growth velocities during pubescence.

To understand the racial difference in mineral acquisition through the major growth years, we have to investigate how black and white children differ in their rates of bone accrual with increasing age as they also transition through different stages of sexual maturation while accounting for earlier transitions in blacks.

Longitudinal data are needed to study growth velocities because when peak velocity occurs at different ages in different children, cross-sectional means of BMC determination in relation to age do not reflect growth rates. We previously compared cross sectionally the BMC of a group of black and white boys and girls(10) who subsequently were followed with annual BMC and Tanner stage measurements for up to 4 years. These data allow us to delineate the effects of chronologic age and Tanner stage on the pattern of bone growth velocities in black and white children, even though the age of transition between Tanner stages differs systematically between the races.

## Subjects and Methods

The subjects were 188 healthy children (91 males, 97 females) who were 5 to 15 years old when they enrolled in the study and had at least two visits. This cohort was a subset of the larger sample whose baseline data were reported earlier as a cross-sectional study of BMC.(10) The sample was recruited through advertisements on campuses, at churches, and in newspapers. The children were healthy and had no history of bone disease or growth problems; they had not taken any medication known to affect bone metabolism. At entry to the study, the age, sex, and self-reported race were recorded. We aimed at recruiting about equal numbers of black and white boys and girls. Informed consent was signed by the children's parents or guardians, with assent obtained from children over 5 years of age. The study was approved by the Institutional Review Board at Indiana University–Purdue University at Indianapolis.

### Study protocol

The study was conducted at the General Clinical Research Center. At baseline and every 12 months, the children made a study visit at which they reported their Tanner stage and had measurements made of their height, weight, bone mineral content, and area. The follow-up ranged from 1 to 4 years.

### Bone measurements

Total-body bone mineral content (TBMC) and area (TAREA) were measured using dual-energy X-ray absorptiometry on a DPXL machine (Lunar Corp., Madison, WI, USA). We did not study areal BMD because it is partially corrected for bone size, which increases during growth, and is therefore difficult to interpret. The short-term measurement error in our laboratory is 1% for TBMC in adults. Long-term stability of the machine was assured by daily measurements of standard external phantoms throughout the course of the study. Subjects were measured on the same instrument throughout the study.

### Body size

Standing height was measured with a wall-mounted stadiometer, and body weight was measured with an electronic digital scale; both scales were calibrated regularly.

### Tanner staging

The children were asked to assess their own sexual development using a sex-specific questionnaire with diagrams of five Tanner stages ranging from stage I (prepubertal) to stage V (fully mature).(16) The research coordinator or a parent then reviewed the results to ensure that the questionnaire was interpreted properly. If a child reported discrepant stages based on two different sexual characteristics, the higher stage was assigned. The validity of self-reported Tanner staging has been established in previous studies.(17–19)

### Statistical methods

Boys and girls have distinct growth patterns because of the earlier time course of sexual maturity in girls. Group means were calculated separately for black and white boys and girls, and all between-race comparisons were made within each sex. The sex-specific results were used to corroborate each other.

To compare with published data, we first plotted the repeated bone measurements (TBMC and TAREA) of individuals against chronologic age. Then we fitted population mean growth curves to the repeated bone measurements using a penalized regression spline method for fitting nonparametric smooth curves(20) separately for black and white boys and girls. However, these fitted curves do not take into account the differences in the Tanner stages at the same chronologic age or the variability in peak growth among individuals.

To study velocities of change in TBMC and TAREA as a function of Tanner stage and chronologic age, we converted each pair of consecutive measurements of each outcome into rates of change [(measurement at second visit – measurement at first visit)/time between visits]. The rate of change per year (annualized change) then was used as the outcome measure of growth velocity in subsequent analyses. For a child with *k* repeated measurements, *k* – 1 velocities were calculated. The effects of Tanner stage and chronologic age on bone growth velocity were first examined separately.

To describe bone growth velocity as a function of Tanner stage alone, means and standard deviations of annualized TBMC and TAREA were calculated in groups classified by the initial Tanner stage, race, and sex. For rates that initiated in Tanner stage I, the sample size was adequate for subdividing into two groups: those at visit 2 who remained in Tanner stage I and those who transitioned into Tanner stage II. All statistical modeling and tests were performed using linear mixed models that included fixed effects of interest and random effects for study subjects to account for multiple annualized changes for some children. Comparison of mean bone growth velocity between black and white children within each Tanner stage was performed for each sex with race as a fixed effect in the mixed model.

Descriptive data for bone growth velocity as a function of age alone were presented as mean growth velocities estimated using the penalized regression spline(20) and plotted against chronologic age for each of the four subgroups of black and white boys and girls.

To control for both the age and sexual-maturation effects, linear mixed models were fitted separately for boys and girls to examine racial differences in bone growth velocity because the children grew in age and advanced in sexual maturation asynchronously. Annualized bone growth velocities were modeled as a function of individuals as random effects, together with chronologic age (polynomial), Tanner stage (discrete), and race, as well as potential interactions among them as fixed effects. These models allowed us to estimate the simultaneous but asynchronous effects of age and sexual maturity separately. The fitted mean velocity curves then were plotted as a function of age within each Tanner stage (from median age of onset to median age of exit) for each subgroup.

The median age of advancing from one Tanner stage to the next was estimated from logistic models fitted separately for each group of black and white boys and girls. For example, to estimate the transition from Tanner stage II to stage III for black boys, all the black boys' Tanner stage measurements were coded as a binary outcome (*Y*) of 1 (stage III and over) or 0 (stage II or under). The binary outcome then was fitted as a logistic function of chronologic age. From the fitted model, the age at which Prob(*Y* = 1) = 0.5 was used as the estimated median age of entry from Tanner stage II to stage III for black boys.

If a significant racial difference in TBMC velocity was detected, either overall or variable according to Tanner stage, then initial height and height velocity, as well as their interactions with Tanner stage, were added to the mixed models for TBMC velocity. If race or race-by-Tanner-stage was still significantly related to TBMC velocity in this model, then the racial difference in TBMC velocity could not be totally attributed to linear growth. The same analyses were repeated with TAREA substituting for height to see if growth in skeletal size explained all of the racial difference in TBMC velocity.

## Results

A total of 188 subjects were included in the study, with an average of 3.3 visits (range 2 to 5) over a mean length of 2.6 years (range 0.9 to 4.6 years). There were 91 boys (39 blacks, 53 whites) and 97 girls (47 blacks, 50 whites), and within each group, the largest subgroup was in Tanner stage I at the start of the study (Table 1).

**Table 1:** Distribution of Age and Tanner Stage of Children at Baseline by Sex and Race

	Boys		Girls	
		
	Black	White	Black	White
*n*	39	52	47	50
Age (years)	10.2 ± 2.7	10.5 ± 2.0	10.6 ± 2.8	10.3 ± 2.6
Tanner stage at baseline				
I	14	26	14	22
II	9	13	7	8
III	7	4	7	7
IV	6	7	6	5
V	3	2	13	8

**Fig. 1:**
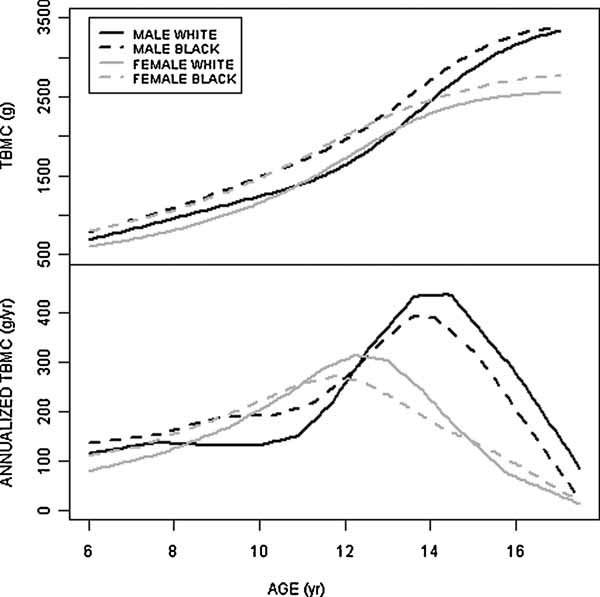
Population mean growth curves of TBMC (*top panel*) with corresponding mean TBMC velocity curves (*bottom panel*) for black and white boys and girls.

**Fig. 2:**
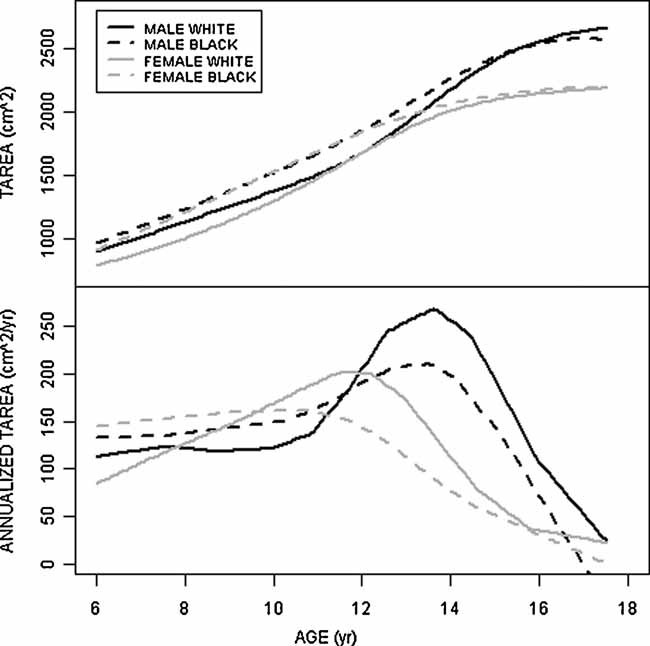
Population mean growth curves of TAREA (*top panel*) with corresponding mean TAREA velocity curves (*bottom panel*) for black and white boys and girls.

Figure 1 (*top panel*) shows the smoothed population mean growth curves of TBMC fitted to all the repeated measurements for each of the four subgroups of black and white boys and girls. Throughout the whole chronologic age range, black children consistently had higher mean TBMC values than white children of the same sex. Boys and girls had similar TBMC values until approximately age 13, after which point the boys had increasingly high TBMC values for the same age. No formal statistical comparisons were made between groups by race or sex. Similar smoothed population velocity curves for TBMC in the bottom panel of Fig. 1 illustrate that even striking changes in velocities with age are not easily discernible from the population growth curve in the top panel. The population growth curves of TAREA (Fig. 2, *top panel*) show a similar pattern to TBMC except that the plateaus converge more between races. Again, the corresponding velocity curves in the bottom panel better reflect changes in rates of growth in TAREA. Thus we performed subsequent analyses to investigate the velocities as measured by the individuals' annualized TBMC and TAREA.

Table 2 summarizes the mean and standard deviation of age and annualized TBMC and TAREA between consecutive visits by race and sex for transitions through different Tanner stages. The groupings were based on the initial Tanner stage, but transitions that started in Tanner stage I were further subdivided into one group that remained in stage I and another group that transitioned to stage II, thus separating the early prepubertal period from the later period when pubertal signs first began to appear. As expected, the black children, on average, were younger than white children of the same sex during every Tanner stage, reaching statistical significance (*p* < .05) only in the highest Tanner stages, as indicated in Table 2. The within-Tanner-stage racial differences in TBMC velocity varied across Tanner stages in a similar pattern between boys and girls. Only the largest differences were statistically significant, as indicated in the table, because of the small subgroup sample sizes, but the overall pattern of racial differences could be discerned. Black children had higher acquisition rates in TBMC than white children all through Tanner stage I, even when they first entered stage II. Once they entered Tanner stage II, however, whites generally had higher TBMC velocities than blacks at the same stage through Tanner stage IV. The highest mean TBMC velocity occurred in Tanner stage IV for boys and in Tanner stage III for girls, implying that individual peak velocities most often occurred in those stages, when white children had higher mean velocity than black children. However, the black children's TBMC velocities surpassed the white children's again after reaching Tanner stage V. These black/white comparisons in Table 2 did not take into account the unequal ages between black and white children within each Tanner stage.

**Table 2:** Mean ± SD of Age and Annualized TBMC and TAREA Velocities Grouped by Transitions Between Tanner Stages by Sex and Race^a^

	Boys		Girls	
		
	Black	White	Black	White
Transition: Tanner stage I to I				
*n*	17	32	18	19
Mean age in interval (years)	7.8 ± 1.3	8.5 ± 1.6	7.2 ± 1.3	7.9 ± 1.5
Annualized TBMC	188.5 ± 81.6	144.7 ± 36.7*	151.8 ± 54.9	115.8 ± 35.7
Annualized TAREA	157.1 ± 55.8	123.2 ± 32.4*	133.0 ± 50.9	114.7 ± 38.2
Transition: Tanner stage I to II				
*n*	4	9	5	10
Mean age in interval (years)	9.5 ± 1.0	9.7 ± 1.1	9.3 ± 2.1	10.4 ± 1.7
Annualized TBMC	201.9 ± 65.6	163.5 ± 24.0	186.4 ± 67.5	181.1 ± 70.3
Annualized TAREA	154.4 ± 37.6	141.2 ± 31.8	172.3 ± 51.4	153.1 ± 60.8
Transition: Tanner stage II to II or III				
*n*	15	18	9	16
Mean age in interval (years)	10.6 ± 1.3	11.4 ± 1.0	10.3 ± 1.5	11.0 ± 1.2
Annualized TBMC	203.1 ± 73.0	210.2 ± 99.6	180.3 ± 60.7	253.9 ± 94.1*
Annualized TAREA	149.4 ± 63.0	182.1 ± 78.7	138.3 ± 54.3	190.7 ± 50.5*
Transition: Tanner stage III to III or IV				
*n*	16	14	16	19
Mean age in interval (years)	12.3 ± 0.9	13.0 ± 0.9*	11.7 ± 1.0	11.6 ± 1.3
Annualized TBMC	269.3 ± 124.0	353.9 ± 71.1*	255.7 ± 101.9	341.9 ± 853*
Annualized TAREA	182.2 ± 73.7	250.3 ± 71.5*	167.1 ± 86.7	230.7 ± 53.9*
Transition: Tanner stage IV to IV or V				
*n*	18	21	22	18
Mean age in interval (years)	13.6 ± 1.0	14.5 ± 1.0*	13.1 ± 1.2	13.4 ± 0.7
Annualized TBMC	403.4 ± 78.6	450.1 ± 136.7	227.6 ± 124.2	247.7 ± 85.0
Annualized TAREA	230.3 ± 77.4	240.5 ± 90.6	122.4 ± 92.1	132.7 ± 50.9
Transition: Tanner stage V to V				
*n*	16	12	44	36
Mean age in interval (years)	14.6 ± 1.2	16.1 ± 1.1*	14.3 ± 1.6	15.0 ± 1.5*
Annualized TBMC	297.4 ± 117.3	189.0 ± 162.3**	147.2 ± 110.4	137.4 ± 103.1
Annualized TAREA	129.2 ± 61.7	92.1 ± 67.3	67.3 ± 75.6	71.1 ± 61.4

a The units of analyses are the intervals between consecutive BMC measurements. Each child may contribute more than one interval in one or more transition categories. Mean age in the interval is the mean of the ages at the beginning and the end of an interval. Age is measured in years, annualized TBMC in grams per year, and annualized TAREA in square centimeters per year.

* Mean significantly different from blacks, *p* < .05.

** Mean different from blacks, *p* < .06.

The estimated median age of entry into each Tanner stage is given with the interquartile range in Table 3. As expected, the median age of transition was consistently lower in girls than in boys and lower in blacks than in whites for all Tanner stages. The total amount of time spent in the pubertal years (Tanner stages II through IV) was longer for boys than for girls by about 1 year. Despite entering puberty at an earlier age, black children spent only 0.2 year longer in puberty compared with white children.

**Table 3:** Estimated Median and Interquartile Range of Age of Entry into Each Tanner Stage

	Boys		Girls	
		
Tanner stage	Black	White	Black	White
Median age (interquartile range) of entry into each Tanner stage (years)				
II	9.4 (8.7–10.2)	10.5 (9.5–11.4)	9.2 (8.5–9.9)	9.7 (8.7–10.7)
III	11.2 (10.5–11.8)	12.3 (11.7–12.9)	10.5 (9.7–11.3)	11.1 (10.1–12.1)
IV	12.3 (11.7–12.8)	13.2 (12.7–13.7)	11.3 (10.4–12.2)	12.4 (11.6–13.1)
V	14.2 (13.4–15.0)	15.1 (14.4–15.8)	12.8 (11.7–14.0)	13.3 (12.6–14.0)
Median length of time elapsed from entry into Tanner stage II to entry into Tanner stage V (years)				
	4.8	4.6	3.8	3.6

Figure 1 (*bottom panel*) shows the TBMC velocities of black and white children as a function of age alone. Within each sex, younger white children generally had lower TBMC velocities than younger black children, whereas the reverse was observed in older children. White children's annualized TBMC values had higher peaks occurring at a later age than black children, but these curves did not take into account the mixture of different Tanner stages at any given age. The TAREA velocities show a similar pattern (Fig. 2, *bottom panel*).

**Fig. 3:**
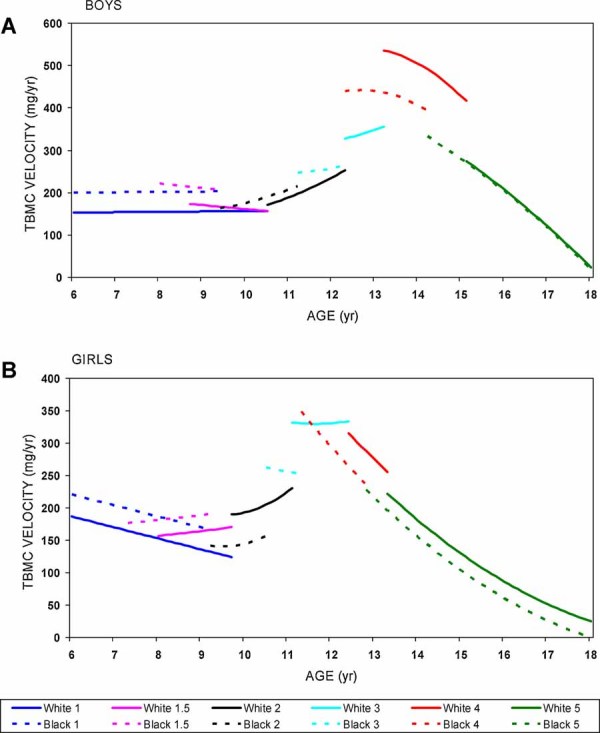
Predicted means of annualized growth velocities of TBMC plotted against age within each Tanner stage transition group. Solid lines are for white children, and dotted lines are for black children, with the same color within each Tanner transition group. The curves for Tanner stages II to IV run from the median age of entry into that stage through the median age of exit. The curves for Tanner stage I span the youngest age in the sample and the median age of exit into Tanner stage II, whereas the curves for Tanner stage V run from the median age of entry to the highest age in the sample.

The linear mixed models used to ascertain the simultaneous effects of chronologic age and sexual maturity on the rate of bone growth showed a significant race-by-Tanner-stage interaction effect on annualized TBMC (*p* = .05 for girls and *p* < .01 for boys) and annualized TAREA (*p* = .06 for girls, *p* < .01 for boys), indicating that the difference in bone growth rates between black and white children varied according to Tanner stage. The predicted velocity of TBMC from the fitted models is plotted against age and Tanner stage separately for boys and girls in Fig. 3.

Figure 3 shows a dynamic picture of how predicted annualized velocities changed with chronologic age as the children advanced through each Tanner stage (from median age of entry to median age of exit). In Fig. 3*A*, younger black boys had higher velocities than white boys in Tanner stage I (group 1 in the graph) through first entry into stage II (group 1.5); the velocity did not change much with age in these stages. After entering into Tanner stage II at an earlier age, black boys maintained a slightly higher age-specific TBMC velocity than white boys through Tanner stage II (group 2). These velocities increased with both chronologic age and advancing Tanner stages through Tanner stage III (group 3), when white boys' age-specific velocities started to overtake those of black boys. Both black and white boys' TBMC velocities peaked at entry into Tanner stage IV (group 4) and then began to decline rapidly with age and progression to Tanner stage V (group 5), when the racial difference was narrowed. The pattern of growth velocities for girls in Fig. 3*B* is similar to that of the boys, except that the white girls' age-specific TBMC velocities overtook those of black girls starting in Tanner stage II, and these velocities peaked around age 11 to 12 regardless of Tanner stage. It is obvious that the higher bone accrual rate in black children occurred primarily in the prepubertal years.

When height velocity was added to these models, it was by far the strongest predictor. Chronologic age was no longer significant and thus was removed. Race-by-Tanner-stage interaction remained predictive but was less significant (*p* = .02 for boys and *p* = .08 for girls) than before height velocity was added. Further addition of initial height and height-by-Tanner-stage interaction in the model reduced the significance of race by Tanner stage (*p* = .05 for boys and *p* = .11 for girls). These results indicated that even after accounting for linear growth and Tanner stage, there was still a racial difference in TBMC velocities that varied across Tanner stages in the boys but not in the girls. However, when initial TAREA and TAREA velocity were substituted for height velocity in the models, there was no more significant racial effect (*p* < .2 for both boys and girls).

When the velocity models in Fig. 3 were integrated over the entire age range of this study to calculate the cumulative bone growth, the estimated population mean TBMC at different ages agreed well with the observed sample means in Fig. 1 (data not shown).

## Discussion

Our population mean curves of TBMC and TAREA (Figs. 1 and 2, *top panels*) agree with all earlier studies that have shown that black children have higher bone mass than white children of the same sex at all ages.(3,9,11–14) Previous studies have found an association between higher peak bone mass and earlier puberty,(21–24) through longer exposure to sex hormones during puberty, as suggested by some.(25–27) Since black children, on average, have earlier puberty than white children, we hypothesized that blacks would have faster bone growth rates and thus greater total bone accrual than whites during puberty. Results of this study, however, show that this rapid growth phase was not the period when black children gained bone faster than whites. Despite earlier entry into puberty (Tanner stage II), black children spent an average of only 0.2 year more in puberty before entry into Tanner stage V compared with white children. Furthermore, black children's age-specific bone growth velocity actually was lower at most stages of rapid bone growth from Tanner stage II to stage IV. Our findings suggest that higher peak bone mass in blacks observed in all studies of adults is not due to earlier puberty or faster bone growth in puberty but is at least partially attributable to their faster bone accrual during the prepubertal years.

To our knowledge, this longitudinal study is the first one to compare bone growth velocities between black and white children, taking into account the simultaneous effects of age and sexual maturation as they occur naturally in a general population. Two other longitudinal studies have compared the growth of TBMC between black and white children. One study found faster growth rates of BMC and BMD in blacks than in whites at ages 8 to 10 years, with the majority of the children in prepuberty,(13) which is in agreement with our findings in Tanner stage I. Another study fitted population mean growth curves to individuals' repeated measurements of BMD as functions of age for different races.(28) It found higher BMD values in blacks at all ages but did not examine individuals' growth velocities. Figures 1 and 2 in our study, however, show that population growth curves (*top panels*) are not suitable for studying growth velocities because even obvious patterns of velocities (*bottom panels*) can be obscured, a shortcoming that is more widely recognized with cross-sectional data.(15)

The complex picture in Fig. 3 shows how TBMC velocity differs between black and white children as both groups advance in chronologic age, but blacks advance through the Tanner stages at younger ages. It underscores the importance of examining the two factors simultaneously over time so that the results are not misleading. For example, mean TBMC velocity was higher in black than in white boys in Tanner stage V (Table 2), when, in fact, their age-specific velocities were almost identical (Fig. 3*A*). Conversely, the higher TBMC velocity of white boys relative to black boys around age 14 to 15 (Fig. 1, *bottom panel*) can be attributed in part to more white boys being in Tanner stage IV (with higher age-specific velocity) and more black boys being in Tanner stage V (with lower age-specific velocity) at that age. Although racial differences can be investigated by matching each white child with a black child of the same sex, age, and Tanner stage, there are two shortcomings. First, the independent contribution of age to the racial difference cannot be assessed. Second, a black child is likely to be slightly more sexually mature than a white child of the same age within a given Tanner stage, which is an imprecise categorization of a continuum of sexual maturity into discrete stages.

We found that most of the growth in TBMC was related to growth in skeletal size in children. All the racial differences in TBMC velocity can be explained by growth in TAREA in both boys and girls. However, while linear growth in height explained most of the racial differences in TBMC velocity in girls, residual differences remain in boys. This may be due to different patterns of bone growth between boys and girls, for example, a different mix of trabecular and cortical bone across pubertal stages,(29) that may affect the relationship between bone mass and size between sexes. Given little black/white differences in adult TAREA but 14% higher TBMC in black men and 8% in black women than in whites of the same sex,(3) it is not surprising that the racial difference in growth of bone mass for size may diverge between the sexes.

Our findings on racial difference in TBMC velocity appear to contradict the findings of Braun and colleagues,(30) who found that adolescent black girls had higher calcium retention than white girls matched on Tanner stage after controlling for the amount of calcium intake. There are several possible reasons for the apparent discrepancy. First, the “adolescent” girls in the calcium balance study ranged from Tanner stage I to V, including pre- and postpubertal girls. Second, the mean calcium intake prior to the study was 654 mg/day (range 202 to 1363 mg/day) for the black girls and 925 mg/day (range 248 to 1741 mg/day) for the white girls. Even at the girls' usual calcium intake levels, however, Braun's model predicts higher calcium retention in the black girls by 118 mg/day, contradicting the higher TBMC velocities observed in our pubertal white girls if a constant proportion of calcium is assumed in total-body mineral. However, statistically controlling for the usual calcium intake requires extrapolating the linear relationship between calcium retention and calcium intake beyond the range of data (700 to 2000 mg/day) down to the lowest intake around 200 mg/day. This may be inappropriate because a nonlinear relationship was observed above an intake of 2000 mg/day, and no data were available below 700 mg/day during the study. Future studies that relate bone growth velocity to calcium retention in black and white children with their usual calcium intake would be needed to resolve these seemingly conflicting findings.

It is important to point out that the velocity model in Fig. 3 does not mean that black children have lower total gain in TBMC from age 5 to 18 years compared with whites. Even though the age-specific rate of bone gain is higher in white children within most Tanner stages above stage I, black children enter into each Tanner stage at an earlier age than white children. During the accelerating growth phase (Tanner stage II through the beginning of Tanner stage IV, when velocities increase with age), advancing Tanner stage has a greater effect on increasing bone growth velocity than the effect of age within a Tanner stage. When black children advance at a younger age to the next Tanner stage during the accelerating growth phase, they enter earlier into a stage with substantially higher age-specific bone growth velocity. During the decelerating phase of bone growth (Tanner stages IV and V), bone growth velocity decreases rapidly with age, but the decrease owing to Tanner stage is smaller. When black children enter into Tanner stages IV and V earlier than white children, more black children benefit from the higher growth velocities at the younger ages within a Tanner stage. Overall, the net gain in TBMC from age 5 to 18 years obtained by integrating the velocities over the ages did not differ substantially between races, implying that the percent difference decreases with age, which agrees with the population growth curves in Fig. 1 (*top panel*). This can be compared with a much larger multisite cross-sectional study,(32) which shows that between the ages of 7 and 16 or 17 years, the absolute black/white difference in TBMC increases to some extent (not detectable in our smaller sample), but the percent difference decreases (from 10% to 5%).

Our study was limited by the age range of the subjects, so we could not determine the onset of racial divergence in TBMC or the timing of complete cessation of bone growth for each race. Not having data prior to age 5, we could not determine when very young black children began to have higher TBMC velocity than whites. Two separate cross-sectional studies in Cincinnati found higher BMC values in black than in white children as young as 1 to 2 years of age(11) but no difference in infants from birth and 18 months of age.(8) Although no age-by-race interaction was detected in the infants, the authors could not rule out a racial difference in bone mineral late in the first year of life because of the limited sample size in the latter study. At the other end of the age range, we could not determine the timing of total cessation of TBMC growth without data beyond age 18, when TBMC continues to grow.(33) We hypothesize that young black adults gain more bone than whites after peak bone size but before peak bone mass is achieved. This conjecture is based on previous findings that while the black/white difference in bone size is small throughout all ages, the 5% difference in TBMC in the late teens(32) increases to 8% (in women) and 14% (in men) by the ages of 25 to 36 years.(3) It is also consistent with little racial difference in cortical volumetric bone mineral density (vBMD = BMC/volume) in children(31) but significantly higher cortical vBMD in black adults.(34) A longitudinal study of young adults of different races starting at age 18 is needed to test whether bone accrual is faster in blacks than in whites after the skeleton has reached full size.

Another limitation of our study is its moderate sample size such that precise estimates for individual subgroups could not be obtained. Nevertheless, the study was sufficiently powered to detect a race-by-Tanner-stage interaction that showed a clear pattern of how black/white differences in TBMC velocity vary as they advance in age and sexual maturation. Given the moderate sample size and the complex pattern of racial differences in this study, we did not investigate potential contributing factors, except that we previously found no significant difference in serum levels of sex hormones between black and white children during puberty.(10) The lower normal calcium intake of black children observed by others(30) potentially could have contributed to their lower peak TBMC velocity in our study. If this is true, the achievable peak bone mass could be even higher in blacks if their calcium intake is increased. However, because peak bone mass is highly heritable,(35) it is likely that much of the higher TBMC in blacks compared with whites is genetically preprogrammed, especially because the same magnitude of difference already exists in early childhood. This is compatible with Bonjour and Chevalley's postulate(36) that pubertal timing and peak bone mass are linked by a common genetic programming rather than through longer exposure to sex hormones with earlier puberty.

In addition to the substantive findings of this study, the analytic methods used here can be applied to other longitudinal comparative studies in which the groups to be compared progress through some states at different rates as the subjects advance in age. The methods here can be used to tease out the effect of age from the effect of progression through states (eg, chronic disease states) on the outcome of interest, and group differences can be estimated as a function of these two factors. For example, one may be interested in racial differences in bone loss as women go through different stages of menopause while they age. Our approach can be used to delineate the effects of aging and of menopause on the rate of bone loss and to compare the rate between races, even if some racial groups transition through the menopausal stages earlier than others.

In summary, we conclude that black children do not differentially gain bone faster than whites during the accelerated bone growth phase in puberty. It remains to be determined when the racial divergence in total bone mineral starts in early childhood and whether differential mineral accretion rates in early adulthood also contribute to racial differences in peak bone mass. Genetic or environmental factors leading to such divergence also need to be explored.
